# Discovery of a novel brown algal genus and species *Setoutiphycus delamareoides* (Phaeophyceae, Ectocarpales) from the Seto Inland Sea, Japan

**DOI:** 10.1038/s41598-021-93320-7

**Published:** 2021-07-06

**Authors:** Hiroshi Kawai, Takeaki Hanyuda

**Affiliations:** Kobe University Research Center for Inland Seas, Rokkodai, Kobe, 657-8501 Japan

**Keywords:** Marine biology, Plant evolution

## Abstract

We describe a new genus and species of brown algae from the Seto Inland Sea, Japan. This species is similar to *Delamarea* in gross morphology and anatomy, but distinctive in having longer thalli with rare branching and shorter cortical cells. In culture, pluri-zoids derived from plurilocular zoidangia on the erect thalli developed into filamentous gametophytes bearing ectocarpoid plurilocular zoidangia, but also formed parenchymatous erect thalli of sub-sympodial growth similar to *Trachynema* often having branches, and formed lateral and terminal plurilocular zoidangia. Molecular phylogenies using concatenated chloroplast and mitochondrial gene sequences showed the new alga nested in the clade composed of ectocarpalean genera with diffuse growth, parenchymatous thalli, and multiple chloroplasts, but this species is distinctive. Therefore, we propose *Setoutiphycus delamareoides* gen. & sp. nov. for this new alga, and provisionally place it in Chordariaceae, Ectocarpales. The Seto Inland Sea repeatedly dried during sea level regressions during glacial periods, and the present sea level recovered after the last glacial maximums (LGM), ca. 10,000 years ago. Therefore, it is unlikely that the species evolved within this area. Its distribution in the area may be explained as a remnant population that survived in refugia in southern Japan during the LGM.

## Introduction

The higher rank taxonomy of brown algae (Phaeophyceae) has been considerably revised in the last few decades, especially in certain orders, by the application of life history studies and molecular phylogenetic analyses^1–5^. Ectocarpales and its related orders, comprising taxonomic groups of relatively small, soft thalli and showing heteromorphic life histories are examples of such revisions. Traditionally, members of Ectocarpales sensu lato (*i.e*., in the broad sense) have been classified in independent orders based on the thallus constructions (filamentous, or pseudoparenchymatous/haplostichous, or parenchymatous/polystichous) and number of chloroplasts per cell (single or multiple). However, life history studies did not suggest distinct boundaries separating these orders, and molecular phylogenetic analyses did not support the monophyly of orders except for the Scytosiphonales. Therefore, it was proposed to classify them in a single order Ectocarpales *s.l.,* including Scytosiphonales as a family (Scytosiphonaceae), and to move many genera that used to be placed in Dictyosiphonales and Chordariales into the family Chordariaceae^6, 7^. As a result, currently more than a hundred genera are included in Chordariaceae, which is an exceptionally large number in the brown algae, and their morphology, including the basic thallus architecture, is highly diverse.

In other phaeophycean orders, the numbers of taxonomically accepted genera range from one (Ascoseirales and Phaeosiphoniellales) to about fifty (Fucales), but mostly less than ten^8^. On the other hand, a new monotypic family has been proposed primarily based on molecular phylogenetic data (i.e., Petrospongiaceae^9^). Also, the taxonomic status of some families is still unclear because their diagnostic characters are not consistent, and phylogenetic resolution of the molecular phylogenetic studies is insufficient. Therefore, family level taxonomic delineation of Ectocarpales is still rather confused and substantial revisions are needed.

Within the Ectocarpales, genus and species level taxonomies of the members with terete, parenchymatous thalli having multiple chloroplasts have been relatively well documented because of their easily recognizable macroscopic thalli, distinctive anatomical features applicable to taxonomic comparisons, and the substantial number of studies using unialgal cultures elucidating their life histories and early development. Most of their genera were described by the 1960’s, and no additional genera have been published for several decades, excluding proposals of a new genus for known species that had been originally described in a different genus^2, 4, 8, 10, 11^. However, recently in the western part of the Seto Inland Sea, Japan (Supplementary Information 1), which has a unique geography but has had only limited taxonomic surveys, we collected an undescribed brown alga with unique morphological features. Here we describe, based on morphology and molecular phylogenetic studies, a new terete parenchymatous species belonging to Ectocarpales *s.l.*, and discuss its family level taxonomy.

## Results

### Morphological studies

**Figure 1:**
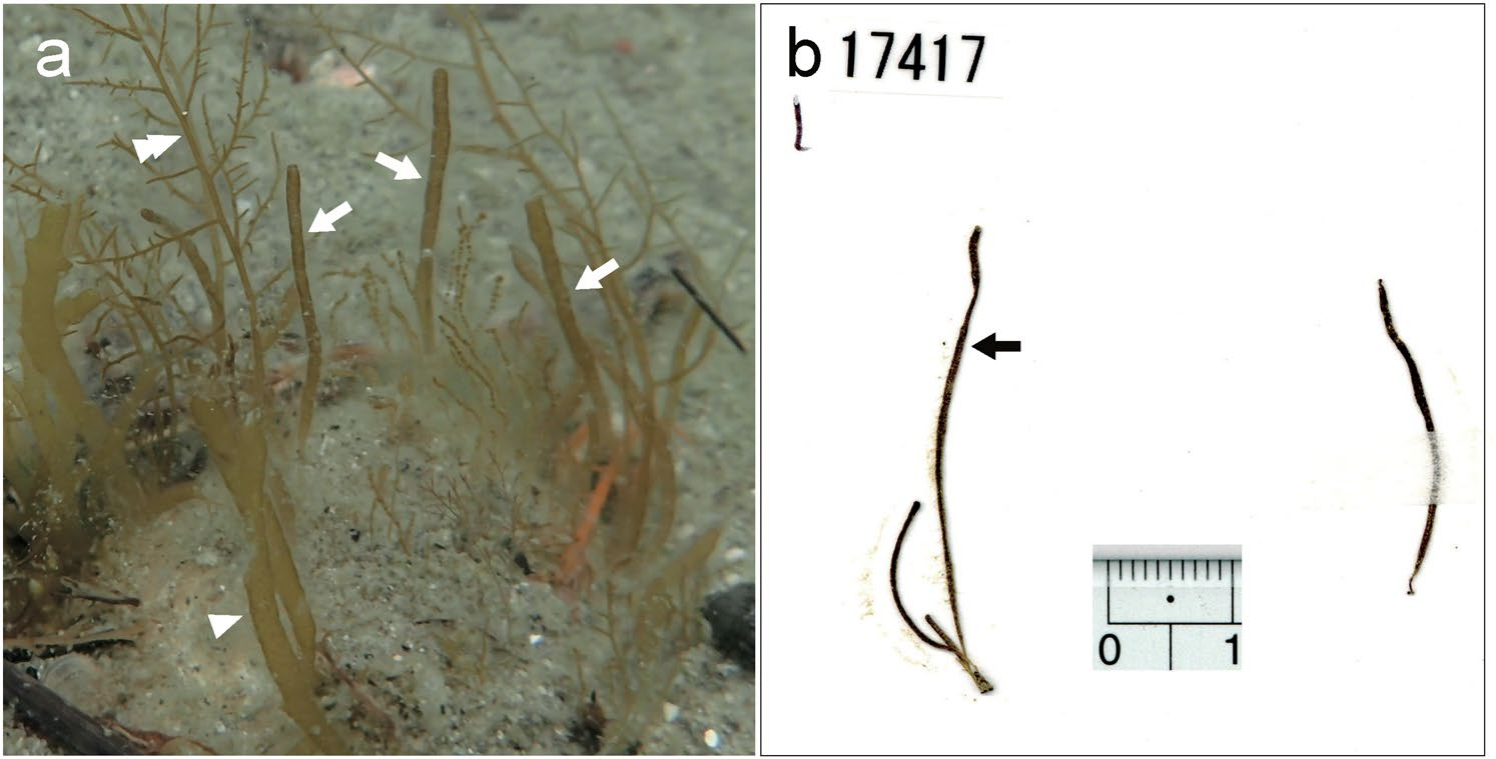
Habit and type specimen of *Setoutiphycus delamareoides* gen. & sp. nov. (Suo-Oshima, 12 April 2017). (**a)** Underwater photograph showing habit of *S. delamareoides* (arrows) growing mixed with *Acrothrix gracilis* (double arrowhead) and *Striaria attenuata* (arrowhead). (**b)** Holotype specimen (arrow; SAP115639; silica gel dried specimen housed as KU-d17417). [Photographs by H. Kawai].

**Figure 2:**
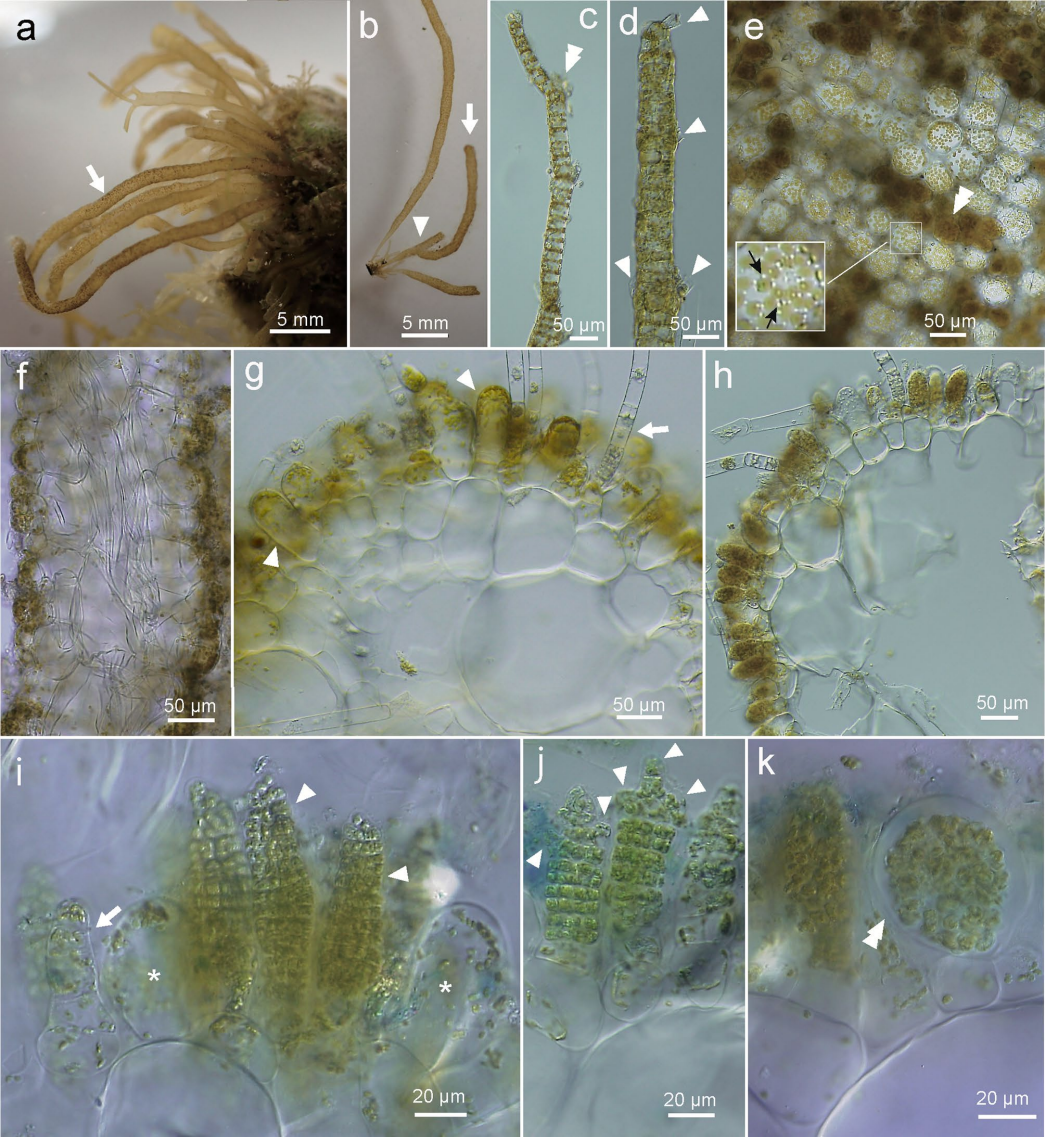
Morphology and anatomy of *Setoutiphycus delamareoides* gen. & sp. nov. (Suo-Oshima, 12 April 2017 and 21 April 2021)*.* (**a**) Erect thalli (arrow) growing on shell of dead barnacle. Reproductive structures are recognizable as dark dots. (**b**) Caespitose erect thalli showing a rare branch (arrowhead) and blunt tip (arrow). (**c**) Young uniseriate erect thallus showing sub-sympodial growth, where elongation of the thallus occurs asymmetrically (double arrowhead). (**d**) Juvenile thallus with apical and lateral (arrowhead) phaeophycean hairs. (**e**) Surface view of fertile erect thallus showing cortical cells containing peripheral discoid chloroplasts with projected pyrenoid (arrows; inset at higher magnification) and plurilocular zoidangia (double arrowhead). (**f)** Longitudinal section showing large inner cells and pigmented cortical cells. (**g**) Cross section of solid thallus showing cylindrical cortical cells (arrowheads) and a phaeophycean hair (arrow). (**h**) Cross section of mature hollow thallus. (**i**) Plurilocular zoidangia (arrowheads) formed among cortical cells (asterisks). Arrow shows immature plurilocular zoidangium. (**j**) Plurilocular zoidangium forming protruding locules at the distal end (arrowheads). (**k**) Unilocular zoidangium (double arrowhead) borne on subcortical cell. (**a**–**h**) Fresh specimens. (**i–k**) Preserved in corn syrup and stained with Cotton Blue. [Photographs by H. Kawai].

This newly found brown alga appeared as a spring annual growing on hard substrates of sandy bottoms of the upper subtidal zone, together with various annuals such as *Acrothrix gracilis* Kylin*, Cutleria multifida* (Turner) Greville, *Striaria attenuata* (Greville) Greville*,* etc. (Fig. 1a). Erect thalli were epilithic or growing on dead shells, solitary or caespitose, filiform, unbranched or rarely branched, attenuated towards the base, blunt at the tip, surface nubby, yellowish brown in color, up to about 15 cm in height and up to 2 mm in diameter (Figs. 1b, 2a, b). The thalli were uniseriate elongating by sub-sympodial growth (Fig. 2c), then becoming parenchymatous and solid when young (Fig. 2d, f, g) and later becoming hollow (Fig. 2h), composed of one to two layers of large colorless inner cells (Fig. 2f–h), subcortical cells (Fig. 2f–h), cylindrical or short clavate cortical cells (Fig. 2f–i), and terminal and lateral phaeophycean hairs (Fig. 2d, g, h). Cortical cells measured up to 100 µm in the long axis and 60 µm in diameter. Inner cells measured up to 250 µm in diameter and subcortical cells measured up to 185 µm in long axis and up to 100 µm in diameter. Plurilocular and unilocular zoidangia were formed on the same thallus, developed among the cortical cells at the distal end of subcortical cells (Fig. 2h–k). Plurilocular zoidangia were formed by subdivisions of cortical cells (Fig. 2i), conical to lanceolate, often with protruding locules at the distal end of well-developed zoidangia (Fig. 2j) and becoming longer than the cortical cells (Fig. 2i), up to 120 µm by 72 µm. Unilocular zoidangia were ovate, up to about 60 µm in long axis and up to 50 µm in diameter (Fig. 2k). Each vegetative cell contained many discoidal chloroplasts with projected pyrenoids (Fig. 2e). In the cortical cells, chloroplasts were parietal towards the surface (Fig. 2g, i).

### Culture studies

**Figure 3:**
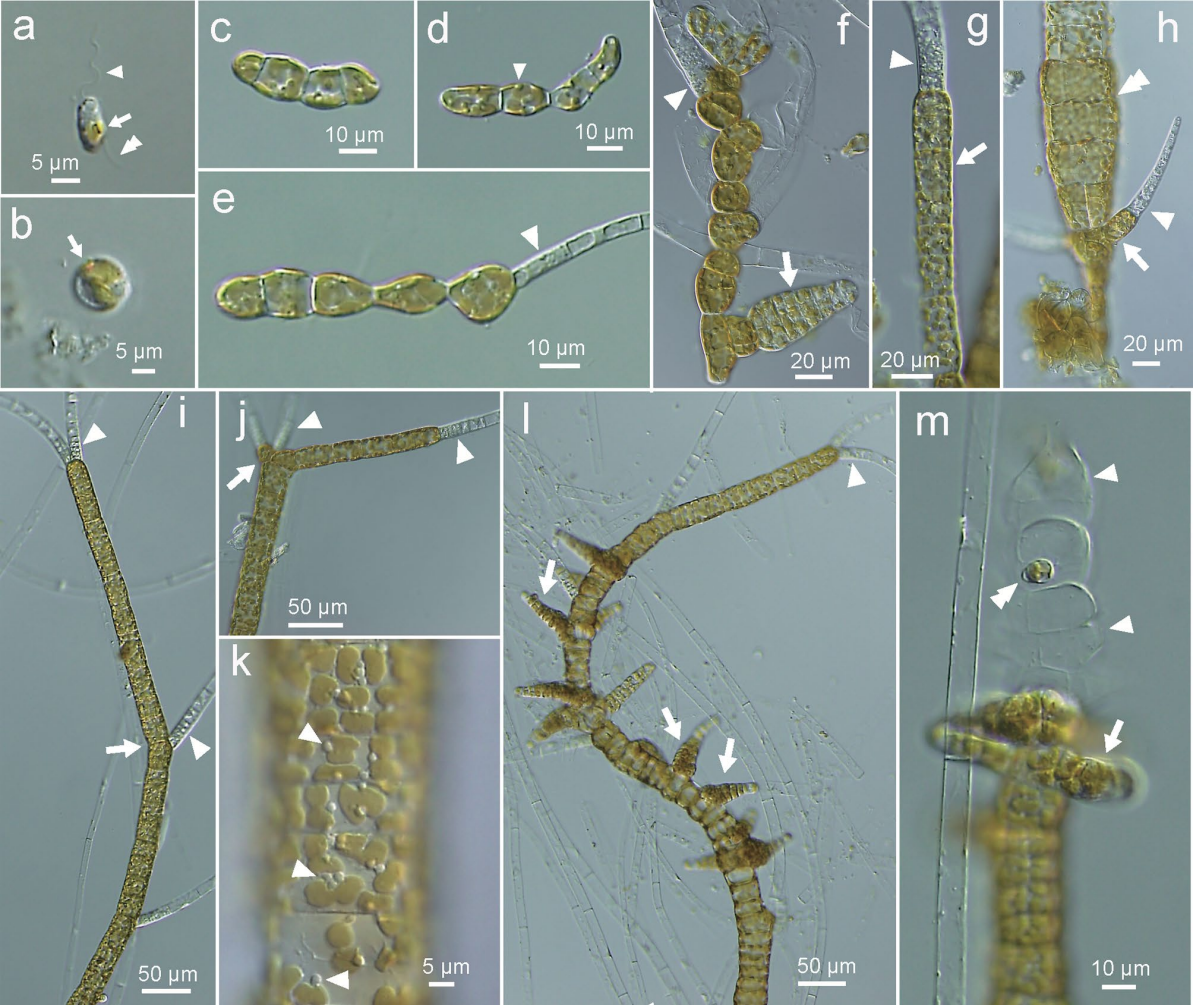
Zoospore, gametophytes and young sporophytes of *Setoutiphycus delamareoides* gen. & sp. nov.. (**a)** Released zoospore. Arrowhead and double arrowhead show anterior and posterior flagella, respectively. Arrow shows stigma. (**b)** Settled zoospore retaining stigma (arrow). (**c–e)** Germlings. Arrowhead shows chloroplast with pyrenoid (**d**). Arrowhead shows phaeophycean hair (**e**). (**f)** Gametophyte forming plurilocular zoidangium (arrow). Arrowhead shows phaeophycean hair. (**g)** Young uniseriate sporophyte (arrow) with terminal hair (arrowhead). (**h)** Young parenchymatous sporophyte (double arrowhead) forming lateral branchlet (arrow) with terminal hair (arrowhead) near the base. (**i)** Young uniseriate sporophyte of sub-sympodial growth (arrow). Arrowheads show phaeophycean hairs. (**j)** Initial of branch (arrow) on young sporophyte. Arrowhead shows phaeophycean hair. (**k)** Surface view of sporophyte showing discoidal chloroplasts with projected pyrenoids (arrowheads). (**l)** Mature sporophyte forming lateral, lanceolate plurilocular zoidangia (arrows). Arrowhead shows terminal phaeophycean hair. (**m)** Plurilocular zoidangia formed at the tip of sporophyte. Arrow shows plurilocular zoidangia. Arrowheads show emplied plurilocular zoidangia. Double arrowhead shows in situ-germinated pluri-zoids. [Photographs by H. Kawai].

Only plurilocular zoidaigia were found on the erect thalli collected on 21 April 2021 and used for the culture studies. Zoids released from plurilocular zoidangia were ca. 10 × 6 µm in size and bi-flagellated with long anterior and short posterior flagella having a stigma on the chloroplast (Fig. 3a). They swam showing negative phototaxis for several minutes and settled on the substrate and formed a cell wall (Fig. 3b). They germinated to develop into a uniseriate filamentous gametophyte and later forming phaeophycean hairs (Fig. 3c–e). Cells of gametophytes contained one to several parietal plate-shaped chloroplasts with projected pyrenoids (Fig. 3c–e). Then the gametophytes formed ectocarpoid plurilocular zoidangia (putative gametangia; Fig. 3f), but also directly formed erect thalli (sporophytes) with terminal phaeophycean hairs (Fig. 3g). The sporophytes developed into simple, parenchymatous erect thalli of sub-sympodial type of growth (Fig. 3i, j), but often they formed lateral and terminal branchlets (Fig. 3h, j). Sporophytic cells contained many disc-shaped chloroplasts with projected pyrenoids (Fig. 3k). When mature, the sporophyte formed ectocapoid plurilocular zoidangia transformed from the cortical cells and uniseriate terminal cells (Fig. 3l, m).

### Molecular phylogeny

**Figure 4:**
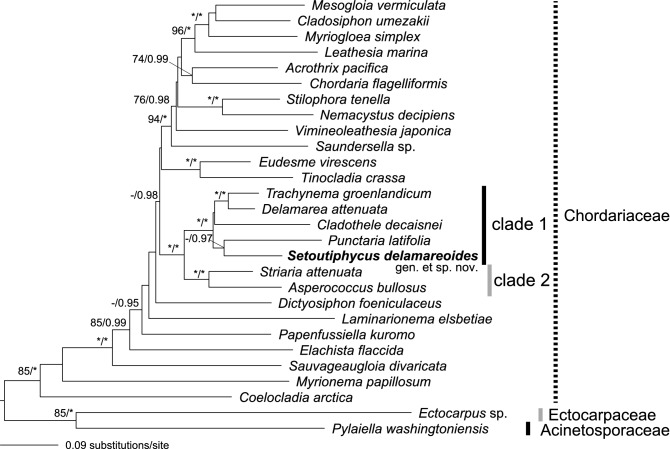
Maximum Likelihood molecular phylogeny of selected ectocarpalean species including *Setoutiphycus delamareoides* gen. & sp. nov. based on concatenated sequences of (*cox*1, *cox*3, *atp*B*, psa*A*, psb*A*, rbc*L genes) (7696 bp). Numbers on branches indicate bootstrap values (%) from ML analysis (left) and posterior probabilities from Bayesian analysis (right). Asterisks (*) indicate 100% bootstrap (ML) and 1.00 posterior probability (Bayesian) values. Only bootstrap values > 70% and posterior probabilities > 0.90 are shown. [Artwork by T. Hanyuda and H. Kawai].

Molecular phylogeny the representative members of Chordariaceae including the new alga (= *Setoutiphycus delamareoides*) based on concatenated mitochondrial *cox*1, *cox*3 and chloroplast *atp*B, *psa*A, *psb*A and *rbc*L gene sequences (7,696 bp) showed identical tree topologies using Maximum Likelihood (ML, Fig. 4) and Bayesian (BI) analyses. The new alga nested in the clade composed of *Trachynema, Delamarea, Cladothele*, and *Punctaria,* supported by full bootstrap/posterior priority values (clade 1), although the supports for the nodes connecting the new alga with other genera were low. Clade 1 was sister to the clade of *Striaria* and *Asperococcus,* supported by full bootstrap/posterior priority values (clade 2). The statistical supports for the node connecting the new alga and *Punctaria* were low (51%) in ML and medium in BI (0.97) analyses.

**Figure 5:**
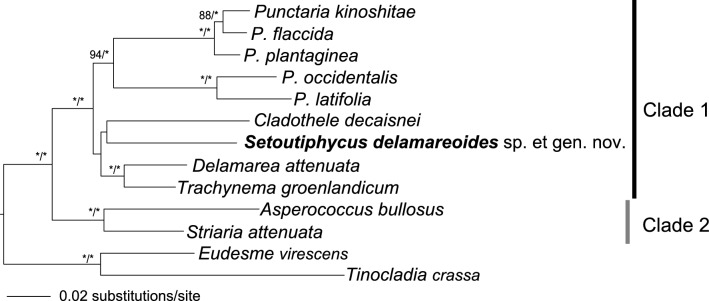
Maximum likelihood molecular phylogeny of *Setoutiphycus delamareoides* gen. & sp. nov. and related taxa based on the concatenated DNA sequences of mitochondrial *cox*1, *cox*3, *nad*2, *nad*5, *nad*6 and chloroplast *atp*B, *psa*A, *psb*A, *psb*A and *rbc*L genes (10,975 bp). Numbers on branches indicate bootstrap (%) from ML and posterior probability values in Bayesian analyses. Asterisk (*) indicates 100% bootstrap and 1.00 posterior probability values. Only bootstrap values ≥ 70% and posterior probabilities ≥ 0.90 are indicated. [Artwork by T. Hanyuda and H. Kawai].

In the ML molecular phylogeny of the taxa comprising clade 1, clade 2 and several representative species of *Punctaria* based on the concatenated DNA sequences of mitochondrial *cox*1, *cox*3, *nad*2, *nad*5, *nad*6 and chloroplast *atp*B, *psa*A, *psb*A and *rbc*L genes (Fig. 5; 10,975 bp), the new alga was sister to *Cladothele* and the clade comprised of the new alga and *Cladothele* was sister to the clade of *Delamarea* and *Trachynema,* although the statistical supports for the nodes were low (45 and 49%, respectively). On the other hand, in BI tree the new alga was sister to the clade of *Punctaria* spp., although the statistical supports were medium to low (0.94 p.p. in BI and < 70% in ML; Supplementary Information 5). Sequence divergences (*P*-distance values) between the new alga and *Punctaria* were 0.018 (*rbc*L) and 0.094 (*cox*3) and were comparable to those among *Asperococcus*, *Cladothele*, *Delamarea*, *Punctaria*, *Striaria* and *Trachynema* (0.010–0.038 in *rbc*L and 0.074–0.101 in *cox*3) (Supplementary Information 6).

**Figure 6:**
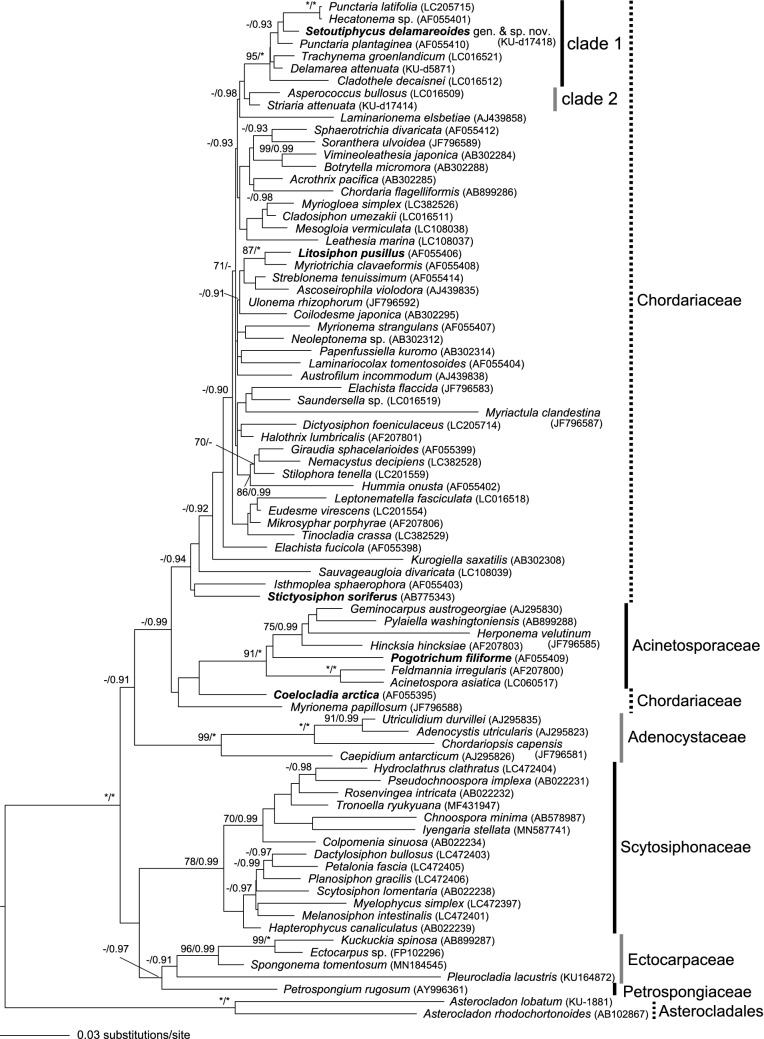
Maximum Likelihood molecular phylogeny of ectocarpacean species including *Setoutiphycus delamareoides* gen. & sp. nov. based on *rbc*L genes sequences (1413 bp). Numbers on branches indicate bootstrap values (%) from ML analysis (left) and posterior probabilities from Bayesian analysis (right). Asterisks (*) indicate 100% bootstrap (ML) and 1.00 posterior probability (Bayesian) values. Only bootstrap values > 70% and posterior probabilities > 0.90 are shown. [Artwork by T. Hanyuda and H. Kawai].

In the molecular phylogeny based on *rbc*L gene sequences covering a large portion of the genera in Ectocarpales, the new alga was also included in the clade composed of *Cladothele*, *Delamarea*, *Hecatonema*, *Punctaria* and *Trachynema,* supported by high bootstrap/p.p. values (95%/1.00), but the relationships among the genera (clade 1) were not resolved (Fig. 6). The new alga was nested in the clade of *Punctaria latifolia* Greville, *P. plantaginea* and *Hecatonema* sp., but the supports for the clade were low. *Hecatonema* has filamentous thalli, but the sequenced specimen was likely a gametophytic stage of *Punctaria* sp.^12^. Clade 1 was sister to clade 2 as in the phylogenetic tree based on six and nine genes, but the statistical support was low. In contrast, some of the ectocarpalean genera such as *Coelocladia*, *Litosiphon*, *Pogotrichum* and *Stictyosiphon*, which have parenchymatous terete thalli and many chloroplasts with projected pyrenoids, and similar basic thallus constructions as the new alga, showed distant phylogenetic relationships with these genera, and were scattered in the *rbc*L tree (Fig. 6; shown in bold-face).

## Discussion

As to the genus level taxonomy of parenchymatous members of Ectocarpales, morphological features of sporophytes such as the gross morphology (simple or branched), growth mode (monopodial or sub-sympodial), thallus constructions (terete or foliose, solid or hollow) and shape of cortical cells and plurilocular zoidangia have been used for defining the genera (Table 1). In morphology, the new alga was most similar to *Delamarea attenuata* Hariot. They shared terete nubby-surfaced thalli with an attenuated basal portion and blunt tip, thallus architecture composed of large inner cells and a cortical layer developing large, short clavate or cylindrical cortical cells, and the occurrence on the erect thalli of both unilocular and plurilocular zoidangia among large cortical cells. However, the new alga was distinctive in having longer thalli of sub-sympodial growth with rare branching, shorter cortical cells, and occurrence of protruding locules at the tips of the mature plurilocular zoidangia. *Cladothele*, *Punctaria*, *Trachynema*, *Striaria* and *Asperococcus*, which showed close phylogenetic relationships in our molecular phylogeny based on the concatenated mitochondrial and chloroplast DNA sequences, share similar thallus morphological features: terete parenchymatous thalli of diffuse growth bearing unilocular and plurilocular zoidangia among large cortical cells, and terminal and lateral phaeophycean hairs.

**Table 1:** Comparisons of representative morphological features between genera in clade 1 in Fig. 5.

	*Setoutiphycus*	*Asperococcus*	*Cladothele*	*Delamarea*	*Punctaria*	*Striaria*	*Trachynema*
Thallus construction	Terete, hollow	Saccate, hollow	Terete, solid	Terete, hollow	Foliose, solid	Terete, hollow	Terete, solid
Growth mode	Sub-sympodial	Monopodial	Monopodial?	Monopodial	Monopodial	Monopodial	Sub-sympodial
Branching	Rarely branched (frequently branched in culture)	Branched	Branched	Unbranched	Unbranched	Branched	Unbranched, (frequently branched in culture)
Cortical cell	Large, cylindrical	Small, globular or polygonal	Large, cylindrical	Large, clavate	Small, globular or polygonal	Small, globular or polygonal	Small, globular or polygonal
Plurilocular zoidangia	Lanceolate, with protruding locules	Lanceolate, no protruding locules	Lanceolate or polygonal, no protruding locules	Lanceolate, no protruding locules	Globular, no protruding locules	Not on macrothallus	Globular or lanceolate, no protruding locules
Unilocular zoidangia	Scattered on entire thallus	Formed in patches with assimilatory filaments	Scattered on entire thallus	Scattered on entire thallus	Scattered on entire thallus	Forming patches with hairs	Scattered on entire thallus
References	This paper	^10, 13^	^14, 15^	^16, 17^	^10, 13, 18^	^10, 19, 20^	^21–23^

In the molecular phylogeny based on nine genes, the new alga was genetically closest to *Cladothele* and *Punctaria* although the statistical supports were low, and the branching order was not clearly resolved. However, they have distinctive morphological features as summarized in Table 1 in the thallus construction, growth mode, branching and the shape of cortical cells. *Cladothele*, *Punctaria* and *Trachynema* have solid thalli, whereas other genera and the new alga have hollow thalli. Although *Punctaria* spp. have foliose thalli, their juvenile thalli show similar terete stages with apical and opposite lateral hairs, which are common to those genera. Among them, only the new alga and *Trachynema* show sub-sympodial type of growth, and perhaps due to the growth mode, although their field-collected thalli are normally simple, they frequently form branches in culture^22^.

In culture, pluri-zoids of the new species developed into uniseriate branched filaments forming ectocarpoid plurilocular zoidangia (gametophyte), but also directly formed erect thalli forming plurilocular zoidangia (sporophyte) which is common in the related genera *Asperococcus*, *Delamarea*, *Punctaria*, *Striaria* and *Trachynema*^13, 16–19, 21–29^. However, among them only *Trachynema* frequently formed branches of the sporophytes, although its natural thalli are simple. Unfortunately, the fate of unizoids were not clarified in the present work because no unilocular zoidangia were found in the new collections. However, dominance of plurilocular zoidangia may be due to the age of the sporophytes, but it is also reported that the ratio of unilocular and plurilocular zoidangia on a thalli may significantly differ depending on individuals in *Delamarea*^17^.

Our multigene molecular phylogeny, including most of the related genera, showed that the new alga is distinct from any of those genera. DNA sequence divergences of *cox*3 gene among representative ectocapalean and laminarialean genera were comparable to the divergence between the new alga and related genera (Supplementary Information 7). Although the phylogenetic relationships with the closely related genera are still not clearly elucidated, probably due to rapid radiation within the Ectocarpales, inclusion of the new alga in any of the existing genera will cause serious confusion in the taxonomy. Therefore, for this new alga we propose the establishment of a new genus and species, *Setoutiphycus delamareoides* gen. & sp. nov*..* In contrast, *rbc*L of other ectocarpalean genera with similar thallus architecture (*e.g.*, *Coelocladia*, *Litosiphon*, *Pogotrichum* and *Stictyosiphon*) showed distant phylogenetic relationships with the clade, suggesting convergent evolution of the thallus architecture in Ectocarpales or maintenance of an ancestral character.

Small brown algae having parenchymatous thalli and multiple chloroplasts with projected pyrenoids have been generally classified in the Dictyosiphonales^24–26^ or Ectocarpales *s.l.*^10, 27^. Within these orders, members were placed in the families Adenocystaceae, Asperococcaceae, Coelocladiaceae, Delamareaceae, Punctariaceae, Striariaceae, etc.^11, 17, 27^. For *Delamarea* and *Cladothele*, a new family Delamareaceae comprised of *Cladothele*, *Coelocladia*, *Delamarea* and *Stschapovia* was proposed based on the anatomical similarity of possessing large cortical cells (paraphyses)^28^. Later, a new order Delamareales was proposed for the family, assuming an isomorphic life history alternating between macrothalli forming plurilocular gametangia (gametophyte) or unilocular zoidangia (sporophyte)^29^. However, *Coelocladia arctica* Rosenvinge and *Delamarea attenuata* were shown by unialgal culture studies to have heteromorphic life histories^30, 31^, and since then the order has not been cited.

Because monophyly of the families with parenchymatous thalli in Dictyosiphonales or Ectocarpales *s.l.* was not supported in molecular phylogenetic studies^6, 32^, expansion of Chordariaceae to include members that used to be classified in independent families such as Punctariaceae and Striariaceae was proposed^6^. In contrast, small brown algae having parenchymatous thalli and a single chloroplast with projected pyrenoids have been classified in Scytosiphonales^33^, and in spite of this unique cytological feature and highly supported monophyly, they are classified as a family nested in Ectocarpales^6, 7^.

However, currently roughly 140 genera are included in Ectocarpales *s.l.,* more than 100 of which are of Chordariaceae, and these numbers are exceptionally large, considering the genetic divergence within each order of the Phaeophyceae^2, 5, 7, 8^ (Supplementary Information 8). The DNA sequence divergence of *rbc*L genes ranges from about 5–15% within each brown algal order. However, divergence was less than 10% in Ectocarpales *s.l.* and not especially high compared with other orders (Supplementary Information 8), which supports the unity of Ectocarpales *s.l.* despite the anomalously large number of genera recognized. In our molecular phylogeny, monophyly of several genera of former members of the Dictyosiphonales (i.e., Delamareaceae [*Delamarea*, *Cladothele*], Punctariaceae [*Punctaria*, *Trachynema*] and Striariaceae [*Striaria*, *Asperococcus*] sharing the following morphological features was supported: Parenchymatous, terete or foliose thalli of diffuse growth, with terminal and lateral (often opposite) phaeophycean hairs; normally forming both unilocular zoidangia and lanceolate to ovoid plurilocular zoidangia on the same thalli; cells with many discoid chloroplasts with projected pyrenoids^34, 35^.

In contrast, in spite of its close morphological similarity with these genera, *Coelocladia* was shown to have only a distant phylogenetic relationship. An independent family Coelocladiaceae has been proposed for *Coelocladia*, based on the unique morphology of the plurilocular zoidangia showing a clustered or crown-like appearance, and the occasional sympodial branching of the primary filament^31^. In contrast, *Dictyosiphon*, the type of Dictyosiphonaceae and Dictyosiphonales, does have parenchymatous terete thalli, but the genus is unique in showing apical growth by a single apical cell, and forming only unilocular zoidangia embedded in the subcortical and peripheral layers^10^. Therefore, based on morphological aspects several independent lineages are recognized, but currently their phylogenetic relationships are not well-resolved. Multigene molecular phylogenies of broader taxa may give clues for evaluating these morphological features and reorganizing their family level taxonomy.

Although the number of taxa we examined was rather limited, our multigene molecular phylogeny based on nine genes showed considerable improvement of the phylogenetic resolution of families within Ectocarpales *s.l.* Therefore, we expect that the application of multigene molecular phylogeny to additional taxa will give clues for obtaining a better taxonomy of the family. As to the taxonomy of the three families traditionally used for the genera comprising the clade including *Setoutiphycus* (*i.e.,* Delamareaceae A.D.Zinova 1953, Punctariaceae (Thuret) Kjellman 1880, Striariaceae Kjellman 1890), Punctariaceae has taxonomic priority. Therefore, it is possible to reappraise Punctariaceae in reorganizing current Chordariaceae by subdividing it into several monophyletic lineages sharing distinctive morphological features. However, for the moment, we suspend any taxonomic treatment, and provisionally place *Setoutiphycus* in Chordariaceae, Ectocarpales *s.l.*

*Setoutiphycus delamareoides* at present is only known from the western end of the Seto Inland Sea, Japan, and it is possibly endemic to the region (Supplementary Information 1). Similarly, an endemic red alga *Neorhodomela enomotoi* Masuda & Kogame was described from the Seto Inland Sea and has not been reported from any other coasts^36^. Members of *Neorhodomela* are cool-temperate or cold-water species, and the localities of the species appear to represent the southern limits of their distributional ranges^37^. In spite of the low latitude (34°N) and short distance from the main flow of the Kuroshio Warm Current, water temperatures at the locality are relatively low (monthly average is from 9.5 to 26.5 °C^38^), because of the enclosed geography of the area. The Seto Inland Sea repeatedly dried due to sea level regression during the glacial periods^39^. Therefore, the establishment of the current macroalgal flora is rather recent, since the present sea level recovered after the last glacial maximum (LGM) of only ca. 10,000 years ago^40^. Therefore, it is unlikely that *Setoutiphycus delamareoides* evolved within this area. It has been noted that the macroalgal flora of the area is more similar to the cool-temperate Pacific coast of northern Honshu (Tohoku region) than that of the adjacent areas in Pacific Shikoku and Kyushu where the water temperature is higher^41^. This similarity is explained as the result of separation of the populations that survived in the refugia in southern Japan during the LGM^42^: During the northward expansion of the populations after the LGM, because of the lower water temperatures in the area, some of them survived in the Seto Inland Sea^39–42^. It is possible that *S*. *delamareoides* has a broader distributional range, at least in Japan, such as more northerly coasts of Honshu, but more sampling is needed to determine this. However, if not, the species can be endangered by the rise of seawater temperature in the area due to global climate change^43, 44^ because the Seto Inland Sea is closed off at its northern end, so the population cannot spread to colder northern coasts.

### Diagnosis

***Setoutiphycus* gen. nov. H. Kawai & Hanyuda.**

Typus: *Setoutiphycus delamareoides* H. Kawai & Hanyuda.

Erect thalli, filiform, rarely branched, attenuated towards the base, blunt at the tip, nubby-surfaced, parenchymatous, solid when young and becoming hollow with age, composed of large colorless inner cells, cylindrical cortical cells, and phaeophycean hairs. Plurilocular and unilocular zoidangia formed on the same thallus at the end of subcortical cells among the large cortical cells. Plurilocular zoidangia conical to lanceolate, often with protruding locules at the distal end. Unilocular zoidangia ovate. Each cell containing many discoidal chloroplasts with projected pyrenoids.

The new genus resembles *Delamarea* in gross morphology and anatomy, but differs in the longer, rarely branched thallus and shorter cortical cells. The species differs from *Cladothele* in the epilithic habit and rare branching, and from *Trachynema* in having large cortical cells. Nucleotide sequences of mitochondrial *cox*1 and *cox*3, chloroplast *atp*B, *psb*A and *rbc*L genes are also distinctive.

***Setoutiphycus delamareoides* sp. nov. H. Kawai & Hanyuda Figs. **1, 2, 3.

Erect thalli, epilithic, solitary or caespitose, filiform, simple or rarely branched, attenuated towards the base, blunt at the tip, nubby-surfaced, yellowish brown in color, up to about 15 cm in height, up to about 2 mm in diameter, parenchymatous, solid when young and becoming hollow with age, composed of 1–2 layers of large colorless inner cells, cylindrical cortical cells, and phaeophycean hairs. Cortical cells measure up to 100 µm in long axis and up to 60 µm in diameter. Inner cells measure up to 250 µm in diameter and subcortical cells measure up to 185 µm in long axis and up to 100 µm in diameter. Plurilocular and unilocular zoidangia on the same thallus, at the end of cortical cells, among the cortical cells. Plurilocular zoidangia conical to lanceolate, often with protruding locules at the distal end, projected from the cortical cells, up to 120 µm in long axis and up to 72 µm in diameter. Unilocular zoidangia ovate, up to ca. 60 µm in long axis and up to ca. 50 µm in diameter. Each cell containing many discoidal chloroplasts with projected pyrenoids. Nucleotide sequences of mitochondrial *cox*1 and *cox*3, chloroplast *atp*B, *psb*A and *rbc*L genes are also distinctive.

*Holotypus*: SAP115639, Suo-Oshima (33.9407 N 132.4016 E), Yamaguchi, Japan, 10, April 2017.

Etymology: The genus name refers to the original locality. The specific epithet refers to the morphological features of the thallus.

## Methods

Specimens were collected at Suo-Oshima, Yamaguchi, Japan on 12 April 2017 and 21 April 2021 by SCUBA diving (Supplementary Information 1). Portions of the specimens were quickly dried in silica gel and used for molecular analyses. For anatomical observations, cross and longitudinal sections were made by hand using a razor blade.

The holotype specimen is housed in the herbarium of the Graduate School of Science, Hokkaido University (SAP). Silica gel-dried specimens (isotypes) are stored in the herbarium of Kobe University Research Center for Inland Seas. For morphological observations, fresh materials and those preserved in corn syrup and stained with Cotton Blue were used. Measurements of cell sizes were made using fresh materials.

Cultures were started from the fertile thalli collected on 21 April 2021. Released zoids were cultured in plastic Petri-dishes containing 15 mL PESI medium^45^. The sets of culture conditions used were 5 °C short day (SD; 8:16 h light:dark), 5 °C long day (LD; 16:8 h light:dark), 10 °C LD, 10 °C SD, 15 °C LD, 15 °C SD, 20 °C LD and 20 °C SD under LED illumination of approximately 30 µmol m^−2^ s^−1^. Unialgal clonal culture strain of *S. delamareoides* are deposited in KU-MACC (Kobe University Macroalgal Culture Collection; KU-####).

DNA extractions were made from field-collected specimens desiccated in silica gel (KU-d4499, -d4519, -d4429, -d4542, -d4564, -d4802, -d5871, -d17414, -d17417, -d17418) and unialgal culture strains housed in the Kobe University Macroalgal Culture Collection (KU-MACC, KU-1881) (Supplementary Information 2). Genomic DNA was extracted using a DNeasy Plant Mini Kit (Qiagen, Hilden, Germany) following the manufacturer’s instructions. Polymerase chain reaction (PCR) amplifications of the mitochondrial *cox*1, *cox*3 and chloroplast *atp*B, *psa*A, *psb*A and *rbc*L were carried out using the KOD FX and KOD FX Neo (ToYoBo, Osaka, Japan) and the TaKaRa PCR Thermal Cycler Dice (Takara Bio, Otsu, Japan). Primers used for PCR and sequencing were as described in Supplementary Information 3. Combinations of the PCR primers were as described in Supplementary Information 4. The profiles of PCRs were as follows: initial denaturation at 94 °C for 2 min; 30–35 cycles of denaturation at 98 °C for 10 s, annealing at 50–46 °C for 30 s, and extension at 68 °C for 30 s; and a final extension at 72 °C for 7 min. After PEG purification^46^, PCR products were sequenced by a DNA sequencing service (FASMAC, Atsugi, Japan). For molecular phylogenetic analyses, published and newly determined DNA sequences were used (Supplementary Information 2). (dataset 1: 28 OTUs, six genes, total 7696 bp; dataset 2: 13 OTUs, nine genes, 10,975 bp dataset 3: 84 OTUs, *rbc*L gene, 1413 bp) were constructed by Maximum Likelihood (ML) and Bayesian analysis (BI). Species of *Ectocarpus* and *Pylaiella* for dataset 1, *Eudesme* and *Tinocladia* for dataset 2, and species of *Asterocladon* for datasets 3 were selected as outgroups. Alignments were made using MAFFT v.6^47^ and then manually adjusted prior to phylogenetic analyses. Molecular phylogenetic trees were constructed by Maximum Likelihood (ML) and Bayesian analysis (BI). RAxML-NG v.1.0.0^48^ was used for ML analyses. The best-fit substitution model for each codon position of each gene was selected by ModelTest-NG v.0.1.7^49^. To find the best tree, 1000 tree searches using 500 random and 500 parsimony-based starting trees for datasets 1&3, 500 tree searches using 250 random and 250 parsimony-based starting trees for datasets 2 were performed. Bayesian analyses were run using MrBayes v.3.2.2^50^. With the aid of the Kakusan4 program^51^, the best-fit substitution model for each codon position of each gene was selected. The Bayesian analyses were initiated with a random starting tree and ran four chains of Markov chain Monte Carlo iterations simultaneously for 10,000,000 generations, keeping one tree every 100 generations. The first 25,000 trees sampled were discarded as ‘burn-in’, based on the stationarity of ln L as assessed using Tracer v.1.7.1^52^. A consensus topology and posterior probability values were calculated from the remaining trees. Genetic distances (p-distance) were calculated using MEGA7^53^.

## Supplementary information

Supplementary Information.
