# Supplementary material for: Discovery of a novel brown algal genus and species Setoutiphycus delamareoides (Phaeophyceae, Ectocarpales) from the Seto Inland Sea, Japan
Source: Sci Rep. 2021 Jul 6;11:13901. doi: 10.1038/s41598-021-93320-7 (PMC8260720; doi:10.1038/s41598-021-93320-7)
Supplement: Supplementary file 1 — Supplementary Information. [file 41598_2021_93320_MOESM1_ESM.pdf]

Supplementary Information

Discovery of a novel brown algal genus and species *Setoutiphycus delamareoides* (Phaeophyceae, Ectocarpales) from the Seto Inland Sea, Japan

Hiroshi Kawai[1,*],Takeaki Hanyuda[1]

[1]Kobe University Research Center for Inland Seas, Rokkodai, Kobe 657-8501, Japan

*kawai@kobe-u.ac.jp

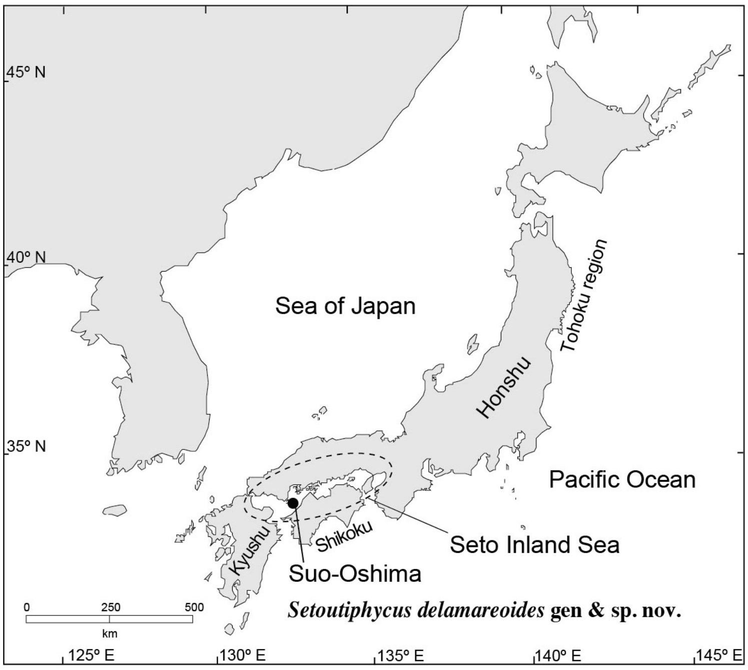

Supplementary Information 1
Map showing the location of the Seto Inland Sea and the collection site of *Setoutiphycus delamareoides* gen. & sp. nov. (Suo-Oshima, Yamaguchi Pref. in the Seto Inland Sea, Japan). The map was drawn by H. Kawai using Adobe Illustrator 2020.

Supplementary Information 2

Origin of specimens and sequence data used for molecular analyses of chloroplast and mitochondrial genes, including their database accession numbers. Sample codes [KU-###] correspond to KU-MACC (Kobe University Macroalgal Culture Collection) strain codes, and [KU-d###] corresponds to silica-gel dried specimens housed at Kobe University Research Center for Inland Seas. Accession codes of sequences newly determined in the present study are indicated in bold.

Species	Origin	Locality	Genes and accession codes								
			*cox*1	*cox*3	*nad*2	*nad*5	*nad*6	*atp*B	*psa*A	*psb*A	*rbc*L
**Asterocladales**											
Asterocladon lobatum	KU-1881	São Paulo, Brazil	-	-				-	-	-	**LC603806**
Asterocladon rhodochortonoides	Uwai *et al.* (2005)	Tsuyazaki, Fukuoka, Japan	-	-				-	-	-	AB102867
**Ectocarpales**											
**Acinetosporaceae**											
Acinetospora asiatica	Yaegashi *et al.* (2015)	Innoshima, Hiroshima, Japan	-	-				-	-	-	LC060517
Feldmannia irregularis	Siemer & Pedersen (only in database)	unknown	-	-				-	-	-	AF207800
Geminocarpus austrogeorgiae	Peters & Ramírez (2001)	unknown	-	-				-	-	-	AJ295830
Herponema velutinum	Silberfeld *et al.* (2014)	unknown	-	-				-	-	-	JF796585
Hincksia hincksiae	Siemer & Pedersen (only in database)	unknown	-	-				-	-	-	AF207803
Pogotrichum filiforme	Siemer *et al.* (1998)	Godthab, Greenland, Denmark	-	-				-	-	-	AF055409
Pylaiella washingtoniensis	Kawai *et al.* (2015)	San Juan I., WA, USA	AB899179	AB526446				AB899197	AB899222	AB899265	AB899288
**Adenocystaceae**											
Adenocystis utricularis	Peters & Ramírez (2001)	unknown	-	-				-	-	-	AJ295823
Caepidium antarcticum	Peters & Ramírez (2001)	unknown	-	-				-	-	-	AJ295826
Chordariopsis capensis	Silberfeld *et al.* (2014)	unknown	-	-				-	-	-	JF796581
Utriculidium durvillei	Peters & Ramírez (2001)	unknown	-	-				-	-	-	AJ295835
**Chordariaceae**											

Acrothrix pacifica	Kawai *et al.* (2016)	Oshoro, Hokkaido, Japan	LC107827	LC107952				LC107972	LC107993	LC108012	-
	Tanaka *et al.* (2010)	Oshoro, Hokkaido, Japan	-	-				-	-	-	AB302285
Ascoseirophila violodora	Peters (2003)	King George I., Antarctica	-	-				-	-	-	AJ439835
Asperococcus bullosus	Kawai *et al.* (2016, 2019a), this study	Ehime, Japan	LC205693	LC016522	**LC636594**	**LC636607**	**LC636623**	LC205701	LC205706	LC205711	LC016509
Austrofilum incommodum	Peters (2003)	King George I., Antarctica	-	-				-	-	-	AJ439838
Botrytella micromora	Tanaka *et al.* (2010)	unknown	-	-				-	-	-	AB302288
Chordaria flagelliformis	Kawai *et al.* (2015, 2019a)	Isle of Man, UK	AB899177	LC205698				AB899195	AB899220	AB899263	AB899286
Cladosiphon umezakii	Kawai *et al.* (2016)	Takeno, Hyogo, Japan	LC107831	LC016526				LC107976	LC107996	LC108016	LC016511
Cladothele decaisnei	Kawai *et al.* (2016), this study	Puerto Williams, Chile	LC107833	LC016527	**LC636595**	**LC636608**	**LC636624**	LC016527	LC107998	LC108018	LC016512
Coelocladia arctica	Kawai *et al.* (2016)	Oshoro, Hokkaido, Japan	LC107834	LC016528				LC107979	LC107999	LC108019	-
	Siemer *et al.* (1998)	Godthab, Greenland, Denmark	-	-				-	-	-	AF055395
Coilodesme japonica	Tanaka *et al.* (2010)	Muroran, Hokkaido, Japan	-	-				-	-	-	AB302295
Delamarea attenuata	KU-d5871	Abashiri, Hokkaido, Japan	**LC603790**	**LC603793**	**LC636596**	**LC636609**	**LC636625**	**LC603797**	**LC603800**	**LC603803**	**LC603807**
Dictyosiphon foeniculaceus	Kawai *et al.* (2019a)	Newfoundland, Canada	-	LC205699				LC205702	LC205707	LC205712	LC205714
	Silberfeld *et al.* (2014)	unknown	JF796539	-				-	-	-	-
Elachista flaccida	Silberfeld *et al.* (2014)	unknown	JF796541	JF796556				JF796631	JF796601	JF796615	JF796583
Elachista fucicola	Siemer *et al.* (1998)	Godthab, Greenland, Denmark	-	-				-	-	-	AF055398
Eudesme virescens	Kawai *et al.* (2019a), this study	Roscoff, Brittany, France	LC201528	LC201560	**LC636597**	**LC636610**	**LC636626**	LC201534	LC201540	LC201546	LC201554
Giraudia sphacelarioides	Siemer *et al.* (1998)	Arrieta, Lanzarote, Spain	-	-				-	-	-	AF055399
Halothrix lumbricalis	Siemer & Pedersen (only on database)	unknown	-	-				-	-	-	AF207801
Haplospora globosa	Siemer *et al.* (1998)	Godhavn, Greenland, Denmark	-	-				-	-	-	AF055400
Hecatonema sp.	Siemer *et al.* (1998)	Hvidøre, Denmark	-	-				-	-	-	AF055401
Hummia onusta	Siemer *et al.* (1998)	Florida, USA	-	-				-	-	-	AF055402

Isthmoplea sphaerophora	Siemer *et al.* (1998)	Godthab, Greenland, Denmark	-	-				-	-	-	AF055403
Kurogiella saxatilis	Tanaka *et al.* (2010)	St. Lawrence I., AK, USA	-	-				-	-	-	AB302308
Laminariocolax tomentosoides	Siemer *et al.* (1998)	Helgoland, Germany	-	-				-	-	-	AF055404
Laminarionema elsbetiae	Kawai *et al.* (2016)	Helgoland, Germany	LC107836	LC016534				LC107982	LC108002	LC108022	-
	Peters (2003)	Helgoland, Germany	-	-				-	-	-	AJ439858
Leathesia marina	Kawai *et al.* (2016)	Ashigezaki, Aomori, Japan	LC107837	LC107964				LC107983	LC108003	LC108023	LC108037
Leptonematella fasciculata	Kawai *et al.* (2016)	Coquimbo, La Herradura, Chile	-	-				-	-	-	LC016518
Litosiphon laminariae	Siemer *et al.* (1998)	Frederikshavn, Denmark	-	-				-	-	-	AF055406
Mesogloia vermiculata	Kawai *et al.* (2016)	Carantec, Brittany, France	LC107839	LC107965				LC107985	LC108005	LC108025	LC108038
Microspongium stilophorae	Siemer *et al.* (1998)	Denmark	-	-				-	-	-	AF055414
Mikrosyphar porphyrae	Siemer & Pedersen (only on database)	unknown	-	-				-	-	-	AF207806
Myriactula clandestina	Silberfeld *et al.* (2014)	unknown	-	-				-	-	-	JF796587
Myriogloea simplex	Kawai *et al.* (2019b), Hanyuda *et al.* 2019)	Ehime, Japan	LC382487	LC382495				LC382503	LC424529	LC382518	LC382526
Myrionema papillosum	Silberfeld *et al.* (2014)	unknown	JF796544	-				JF796636	JF796605	JF796620	JF796588
Myrionema strangulans	Siemer *et al.* (1998)	Frederikshavn, Denmark	-	-				-	-	-	AF055407
Myriotrichia clavaeformis	Siemer *et al.* (1998)	Frederikshavn, Denmark	-	-				-	-	-	AF055408
Nemacystus decipiens	Kawai *et al.* (2019b)	Ishikawa, Japan	LC382489	LC382497				LC382505	LC382512	LC382520	LC382528
Neoleptonema sp.	Tanaka *et al.* (2010)	Shimoda, Shizuoka, Japan	-	-				-	-	-	AB302312
Papenfussiella kuromo	Kawai *et al.* (2016)	Ofunato, Iwate, Japan	LC107840	LC107966				LC107986	LC108006	LC108026	-
	Tanaka *et al.* (2010)	Fukaura, Aomori, Japan	-	-				-	-	-	AB302314
Punctaria flaccida	KU-d4499	Funekawa, Akita, Japan	**LC618935**	**LC618942**	**LC636598**	**LC636611, LC636612**	**LC636627**	**LC636635**	-	**LC636639**	**LC618949**
Punctaria kinoshitae	KU-d4564	Asamushi, Aomori, Japan	**LC618936**	**LC618943**	**LC636599**	**LC636613**	**LC636628**	**LC636636**	-	**LC636640**	**LC618950**
Punctaria latifolia	Kawai *et al.* (2019a)	Pardelas, Argentina	LC205696	LC205700	**LC636600**	**LC636614, LC636615**	**LC636629**	LC205704	LC205710	LC205713	LC205715

Punctaria occidentalis	KU-d4752	Usu, Hokkaido, Japan	**LC618937**	**LC618944**	**LC636601**	**LC636616**	**LC636630**	-	-	**LC636641**	**LC618951**
Punctaria plantaginea	Siemer *et al.* (1998)	Godthab, Greenland, Denmark	-	-	-	-	-	-	-	-	AF055410
	KU-d4542	Fukaura, Aomori, Japan	**LC618938**	**LC618945**	**LC636602**	**LC636617, LC636618**	**LC636631**	**LC636637**	**LC636638**	**LC636642**	**LC618952**
Saundersella crassa	Kawai *et al.* (2016)	Abashiri, Hokkaido, Japan	LC107841	LC016536	-	-	-	LC107987	LC108007	LC108027	LC016519
Sauvageaugloia divaricata	Kawai *et al.* (2016)	Porz Liogan, Brittany, France	LC107842	LC107967				LC107988	LC108008	LC108028	LC108039
***Setoutiphycus delamareoides* gen. & sp. nov.**	Isotype: KU-d17418	Suo-Oshima, Yamaguchi, Japan	**LC603791**	**LC603794**	**LC636603**	**LC636619**	**LC636632**	**LC603798**	**LC603801**	**LC603804**	**LC603808**
	Holotype: SAP115639 (=KU-d17417)	Suo-Oshima, Yamaguchi, Japan	-	**LC603795**	-	-	-	-	-	-	-
Soranthera ulvoidea	Silberfeld *et al.* (2014)	unknown	-	-	-	-	-	-	-	-	JF796589
Sphaerotrichia divaricata	Siemer *et al.* (1998)	Falshoft, Gelting, Germany	-	-	-	-	-	-	-	-	AF055412
Stictyosiphon soriferus	Kawai *et al.* (2013)	Bergen, Norway	-	-	-	-	-	-	-	-	AB775343
Stilophora tenella	Kawai *et al.* (2019a)	Dorset, England, UK	LC201533	LC201584	-	-	-	LC205705	LC201545	LC201553	LC201559
Striaria attenuata	KU-d17414	Suo-Oshima, Yamaguchi, Japan	**LC603792**	**LC603796**	**LC636604**	**LC636620**	**LC636633**	**LC603799**	**LC603802**	**LC603805**	**LC603809**
Tinocladia crassa	Kawai *et al.* (2016, 2019a)	Nomozaki, Nagaki, Japan	LC205697	LC107970	**LC636605**	**LC636621**	-	LC107990	LC108009	LC108030	LC382529
Trachynema groenlandicum	Kawai *et al.* (2016)	Muroran, Hokkaido, Japan	LC107844	LC016539	**LC636606**	**LC636622**	**LC636634**	LC107991	LC108010	LC108031	LC016521
Ulonema rhizophorum	Silberfeld *et al.* (2014)	unknown	-	-				-	-	-	JF796592
Vimineoleathesia japonica	Kawai *et al.* (2016)	Shimamaki, Hokkaido, Japan	LC107845	LC107971				LC107992	LC108011	LC108032	-
	Tanaka *et al.* (2010)	Shimamaki, Hokkaido, Japan	-	-				-	-	-	AB302284
**Ectocarpaceae**											
Ectocarpus sp.	Cock *et al.* (2010)	San Juan de Marcona, Peru	FP885846	FP885846				-	-	-	-
	Le Corguille *et al.* (2009)	San Juan de Marcona, Peru	-	-				FP102296	FP102296	FP102296	FP102296
Kuckuckia spinosa	Kawai *et al.* (2015)	Villefranche-sur-Mer, France	-	-				-	-	-	AB899287
Pleurocladia lacustris	SAG 25.93; Wang *et al.* (only in database)	River Fischa-Dagnitz, Austria	-	-				-	-	-	KU164872
Spongonema tomentosum	Bringloe *et al.* (2019)	Hordaland, Norway	-	-				-	-	-	MN184545

**Petrospongiaceae**								
Petrospongium rugosum	Cho & Boo (2006)	Jejudo, Korea	-	-	-	-	-	AY996361
**Scytosiphonaceae**								
Chnoospora minima	Kogame *et al.* (2011)	Lanai, HI, USA	-	-	-	-	-	AB578987
Colpomenia sinuosa	Kogame *et al.* (1999)	Kasumi, Hyogo, Japan	-	-	-	-	-	AB022234
Dactylosiphon bullosus	Hanyuda *et al.* (2020)	Kirikiri, Iwate, Japan	-	-	-	-	-	LC472403
Hapterophycus canaliculatus	Kogame *et al.* (1999)	Oshoro, Hokkaido, Japan	-	-	-	-	-	AB022239
Hydroclathrus clathratus	Hanyuda *et al.* (2020)	Shibushi, Kagoshima, Japan	-	-	-	-	-	LC472404
Iyengaria stellata	Santiañez *et al.* (2020)	Doha, Kuwait Bay, Kuwait	-	-	-	-	-	MN587741
Melanosiphon intestinalis	Hanyuda et al. (2020)	Shizunai, Hokkaido, Japan	-	-	-	-	-	LC472401
Myelophycus simplex	Hanyuda *et al.* (2020)	Kada, Wakayama, Japan	-	-	-	-	-	LC472397
Petalonia fascia	Hanyuda *et al.* (2020)	Kamaishi, Iwate, Japan	-	-	-	-	-	LC472405
Planosiphon gracilis	Hanyuda *et al.* (2020)	Misaki, Osaka, Japan	-	-	-	-	-	LC472406
Pseudochnoospora implexa	Kogame *et al.* (1999)	Sesoko, Okinawa, Japan	-	-	-	-	-	AB022231
Rosenvingea intricata	Kogame *et al.* (1999)	Gushikawa, Okinawa, Japan	-	-	-	-	-	AB022232
Scytosiphon lomentaria	Kogame *et al.* (1999)	Oshoro, Hokkaido, Japan	-	-	-	-	-	AB022238
Tronoella ryukyuana	Santiañez *et al.* (2020)	Itoman, Okinawa, Japan	-	-	-	-	-	MF431947

References of Supplementary Information 2

Bringloe, T. T., Sjotun, K. & Saunders, G. W. A DNA barcode survey of marine macroalgae from Bergen (Norway). *Mar. Biol. Res.* **15**, 580–589 (2019).

Cho, G. Y. & Boo, S. M. Phylogenetic position of *Petrospongium rugosum* (Ectocarpales, Phaeophyceae): insights from the protein-coding plastid *rbc*L and *psa*A gene sequences. *Cryptogam. Algol.* **27**, 3–15 (2006).

Cock, J. M., Sterck, L., Rouze, P. *et al.* The *Ectocarpus* genome and the independent evolution of multicellularity in brown algae. *Nature* **465**, 617–621 (2010).

Hanyuda, T., Takeuchi, K. & Kawai, H. *Tinocladia sanrikuensis* sp. nov. (Ectocarpales *s.l.*, Phaeophyceae) from Japan. *Phycol. Res.* **67**, 221–227 (2019).

Hanyuda, T., Aoki, S. & Kawai, H. Reinstatement of *Myelophycus caespitosus* (Scytosiphonaceae, Phaeophyceae) from Japan. *Phycol. Re*s. **68**, 126–134 (2020).

Kawai, H., Hanyuda, T., Ridgway, L. M. & Holser, K. Ancestral reproductive structure in basal kelp *Aureophycus aleuticus*. *Sci. Rep.* **3**, 2491 (2013).

Kawai, H., Hanyuda, T., Draisma, S. G., Wilce, R. T. & Andersen, R. A. Molecular phylogeny of two unusual brown algae, *Phaeostrophion irregulare* and *Platysiphon glacialis*, Stschapoviales ord. nov. and Platysiphonaceae fam. nov., and a re-examination of divergence times for brown algal orders. *J. Phycol.* **51**, 918–928 (2015).

Kawai, H., Hanyuda, T., Kim, S.-H., Ichikawa, Y., Uwai, S. & Peters, A. F. *Cladosiphon takenoensis* sp. nov. (Ectocarpales s.l., Phaeophyceae) from Japan. *Phycol. Res.* **64**, 212–218 (2016).

Kawai, H., Hanyuda, T., Sun, Z., Barbara, I. & Peters, A. F. Taxonomic revision of *Eudesme* (Ectocarpales s.l., Phaeophyceae) proposing a new species *E. borealis* sp. nov. *Phycologia* **58**, 351–358 (2019a).

Kawai, H., Hanyuda, T., Shibata, K., Kamiya, M. & Peters, A. F. Proposal of a new brown algal species, *Mesogloia japonica* sp. nov. (Chordariaceae, Phaeophyceae), and transfer of *Sauvageaugloia ikomae* to *Mesogloia*. *Phycologia* **58**, 63–69 (2019b).

Kogame, K., Horiguchi, T. & Masuda, M. Phylogeny of the order Scytosiphonales (Phaeophyceae) based on DNA sequences of *rbc*L, partial *rbc*S, and partial LSU nrDNA. *Phycologia* **38**, 496–502 (1999).

Le Corguillé, G., Pearson, G., Valente, M. *et al.* Plastid genomes of two brown algae, *Ectocarpus siliculosus* and *Fucus vesiculosus*: further insights on the evolution of red-algal derived plastids. *BMC Evol. Biol.* **9**, 253–266 (2009).

Peters, A. F. Molecular identification, distribution and taxonomy of brown algal endophytes, with emphasis on species from Antarctica. *Proc. Int. Seaweed Symp.* **17**, 293–302. (2003).

Peters, A. F. & Ramírez, M. E. Molecular phylogeny of small brown algae, with special reference to the systematic position of *Caepidium antarcticum* (Adenocystaceae, Ectocarpales). *Cryptogam. Algol.* **22**, 187–200 (2001).

Santiañez, W. J. E., Lee, K. M., Uwai, S., Kurihara, A., Geraldino, P. J. L., Ganzon-Fortes, E. T., Boo, S. M. & Kogame, K. Untangling nets: elucidating the diversity and phylogeny of the clathrate brown algal genus *Hydroclathrus*, with the description of a new genus *Tronoella* (Scytosiphonaceae, Phaeophyceae). *Phycologia* **57**, 61–78 (2020).

Siemer, B. L., Stam, W. T., Olsen, J. L. & Pedersen, P. M. Phylogenetic relationships of the brown algal orders Ectocarpales, Chordariales, Dictyosiphonales, and Tilopteridales (Phaeophyceae) based on Rubisco large subunit and spacer sequences. *J. Phycol.* **34**, 1038–1048 (1998).

Silberfeld, T., Rousseau, F. & de Reviers, B. An updated classification of brown algae (Ochrophyta, Phaeophyceae). *Cryptogam. Algol.* **35**, 117–156 (2014).

Tanaka, A., Uwai, S., Nelson, W. & Kawai, H. *Phaeophysema* gen. nov. and *Vimineoleathesia* gen. nov., new brown algal genera for the minute Japanese members of the genus *Leathesia*. *Eur. J. Phycol.* **45**, 109–117 (2010).

Uwai, S., Nagasato, C., Motomura, T. & Kogame, K. Life history and molecular phylogenetic relationships of *Asterocladon interjectum* sp. nov. (Phaeophyceae). *Eur. J. Phycol.* **40**, 179–194 (2005).

Yaegashi, K., Yamagishi, Y., Uwai, S., Abe, T., Santianez, W. J. E. & Kogame, K. Two species of the genus *Acinetospora* (Ectocarpales, Phaeophyceae) from Japan: *A. filamentosa* comb. nov. and *A. asiatica* sp. nov. *Bot. Mar.* **58**, 331–343 (2015).

Supplementary information 3

List of primers used for polymerase chain reaction (PCR) and DNA sequencing.

Code	F/R	Sequence (5'-3')	Region	Reference
ycf3-F1P	F	CAAGCDYTAAATAATATWGCTG	*ycf*3	Kawai *et al*. (2013)
atpB-F1.3P	F	GTHMGHGCNATTGCNATGAGTGC	*atp*B	Kawai *et al*. (2013)
atpB-2R	R	AGCTTGWACAAATCTAAAAATA	*atp*B	Silberfeld *et al*. (2010)
atpB-R1P	R	TTTGCTTTAGMDATWGCTTC	*atp*B	Kawai *et al*. (2013)
psaA130F	F	AACWACWACTTGGATTTGGAA	*psa*A	Yoon *et al*. (2002)
psaA970R2	R	TRCTATGDCCRATNCCCCAA	*psa*A	Kawai *et al*. (2013)
psaA1760R2	R	CCRTCACAHGGRAAWCGGAA	*psa*A	Kawai *et al*. (2013)
psaA1760R	R	CCTCTWCCWGGWCCATCRCAWGG	*psa*A	Yoon *et al*. (2002)
psaA-P4	R	NGATTCAATHARYTCTTGCC	*psa*A	Kawai *et al*. (2012)
psbA-F2	F	GCAACTTTAGAAAGACGCGA	*psb*A	Yoon *et al*. (2002)
psbA500F	F	CTCTGATGGWATGCCWYTAGG	*psb*A	Yoon *et al*. (2002)
psbA600R	R	CCAAATACACCAGCAACACC	*psb*A	Yoon *et al*. (2002)
psbA-R1.2	R	YYWGAWGCTAAATCTAATGGG	*psb*A	Kawai *et al*. (2018)
rbcL-P2	F	GAWCGRACTCGAWTWAAAAGTG	*rbc*L	Kawai *et al*. (2007)
rbcL-P2.2	F	CTCGAWTWAAAAGTGAVCGWTAYGAATC	*rbc*L	Kawai *et al*. (2012)
rbcL-Rh3	F	TTAAYTCTCARCCDTTYATGCG	*rbc*L	Hanyuda *et al*. (2004)
Ral-R952	R	CATACGCATCCATTTACA	*rbc*L	Kawai *et al*. (2007)
rbcS-P1	R	GGATCATCTGYCCATTCTACAC	*rbc*S	Kawai *et al*. (2007)
nad2-P1	F	CDGCHCCHTAYCAYATGTGG	*nad*2	Kawai *et al*. (2017)
nad2-P1.2	F	CCHTAYCATATGTGGATAGC	*nad*2	This study
GazF2	F	CCAACCAYAAAGATATWGGTAC	*cox*1	Lane *et al*. (2007)
cox1-P1.2	F	GATHTTYTTTATGGTDATGCC	*cox*1	Kawai *et al*. (2013)
cox1-P6	R	CCAAAARCTWATRTTATTCAT	*cox*1	Kawai *et al*. (2017)
GazR2	R	GGATGACCAAARAACCAAA	*cox*1	Lane *et al*. (2007)
cox1-P3	R	CNGTAAACATRTGRTGVGCC	*cox*1	Kawai *et al*. (2013)
cox1-P2	R	GGDATACGDCGHGGCATACC	*cox*1	Kawai *et al*. (2013)
trnI-P1.3	R	ACACTCTACCRCTGAGTTAC	*trn*I	This study
trnI-P1.2	R	GCTTATCAGGCGTACACTCT	*trn*I	Kawai *et al*. (2013)
trnI-P1	R	TTGAACGAWCGVCTTTACGC	*trn*I	Kawai *et al*. (2013)
trnY-P2	F	GKCAGATTGTAAATCTGTTGG	*trn*Y	Kawai *et al*. (2012)
trnY-P1	F	TCYATCRTAGGTTCGAATCC	*cox*3	Ni-Ni-Win *et al*. (2008)
cox3-P2	R	ACAAARTGCCAATACCAAGC	*cox*3	Ni-Ni-Win *et al*. (2008)
nad4-P1	F	ACHYTKDTHATGGGAATTTATCC	*nad*4	Kawai *et al*. (2017)
nad5-P1	F	GGYCTTATHGARAGTTTAGG	*nad*5	Kawai *et al*. (2017)
nad5-P1.2	F	GARAGTTTWGGDCCYTTTGG	*nad*5	This study
nad5-P2	R	CTRTCRAARCAAAAMSCCCAATC	*nad*5	Kawai *et al*. (2017)

References of Supplementary information 3.

Hanyuda, T., Suzawa, Y., Suzawa, T., Arai, S., Sato, H., Ueda, K. & Kumano, S. Biogeography and taxonomy of *Batrachospermum helminthosum* Bory (Batrachospermales, Rhodophyta) in Japan inferred from *rbc*L gene sequences. *J. Phycol.* **40**, 581–588 (2004).

Kawai, H., Hanyuda, T., Draisma, S. G. A. & Müller, D. G. 2007. Molecular phylogeny of *Discosporangium mesarthrocarpum* (Phaeophyceae) with a reassessment of the Discosporangiales. *J. Phycol.* **43**, 186–194 (2007).

Kawai, H., Hanyuda, T., Ridgway, L. M. & Holser, K. Ancestral reproductive structure in basal kelp *Aureophycus aleuticus*. *Sci. Rep*. **3**, 2491 (2013).

Kawai, H., Hanyuda, T., Gao, X., Terauchi, M., Miyata, M., Lindstrom, S. C., Klochkova, N. G. & Miller, K. A. Taxonomic revision of the Agaraceae with a description of *Neoagarum* gen. nov. and reinstatement of *Thalassiophyllum*. *J. Phycol*. **53**, 261–270 (2017).

Kawai, H., Kogishi, K., Hanyuda, T. & Kitayama, T. Taxonomic revision of the genus *Cutleria* proposing a new genus *Mutimo* to accommodate *M. cylindrica* (Cutleriaceae, Phaeophyceae). *Phycol. Res*. **60**, 241–248 (2012).

Lane, C. E., Lindstrom, S. C. & Saunders, G. W. A molecular assessment of northeast Pacific *Alaria* species (Laminariales, Phaeophyceae) with reference to the utility of DNA barcoding. *Mol. Phylog. Evol.* **44**, 634–648 (2007).

Ni-Ni-Win, Hanyuda, T., Arai, S., Uchimura, M., Abbott, I. A. & Kawai, H. Three new records of *Padina* in Japan based on morphological and molecular markers. *Phycol. Res*. **56**, 288–300 (2008).

Silberfeld, T., Leigh, J. W., Verbruggen, H., Cruaud, C., de Reviers, B. & Rousseau, F. 2010. A multi-locus time-calibrated phylogeny of the brown algae (Heterokonta, Ochrophyta, Phaeophyceae): investigating the evolutionary nature of the "brown algal crown radiation". *Mol. Phylogen. Evol*. **56**, 659–674 (2010).

Yoon, H. S., Hackett, J. D. & Bhattacharya, D. A single origin of the peridinin–and fucoxanthin-containing plastids in dinoflagellates through tertiary endosymbiosis. *Proc. Natl. Acad. Sci. U.S.A.* **99**, 11724–11729 (2002).

Supplementary information 4.

Combination of primers for 1st and 2nd PCR.

Gene	1st PCR	2nd PCR (parts of gene)
*atp*B	ycf3-F1P, atpB-R1P	atpB-F1.3P, atpB-R1P (entire region)
		atpB-F1.3P, atpB-2R (5' half)
		ycf3-F1P, atpB-2R (5' half)
*psa*A	psaA130F, psaA-P4	psaA130F, psaA1760R (entire region)
*psb*A	psbA-F2, psbA-R1.2	psbA-F2, psbA-R1.2 (entire region)
*rbc*L	rbcL-P2, rbcS-P1	rbcL-P2, Ral-R952 (5' half)
		rbcL-Rh3, rbcS-P1 (3' half)
*cox*1	GazF2, trnI-P1	GazF2, trnI-P1 (entire region)
		GazF2, GazR2 (5' half)
	nad2-P1, GazR2	nad2-P1, cox1-P6 (5' half)
*cox*3	trnY-P2, cox3-P2	trnY-P1, cox3-P2 (entire region)
*nad*2	nad2-P1, cox1-P3	nad2-P1, GazR2 (3' half)
*nad*5	nad4-P1, nad5-P8	nad4-P1, nad5-P8 (5' half)
		nad4-P1, nad5-P4.2 (5' half)
	nad5-P6, nad6-P3	nad5-P6, nad6-P3 (3' half)
*nad*6	nad5-P1, nad11-P1	nad5-P1, nad11-P1 (entire region)

Supplementary Information 5

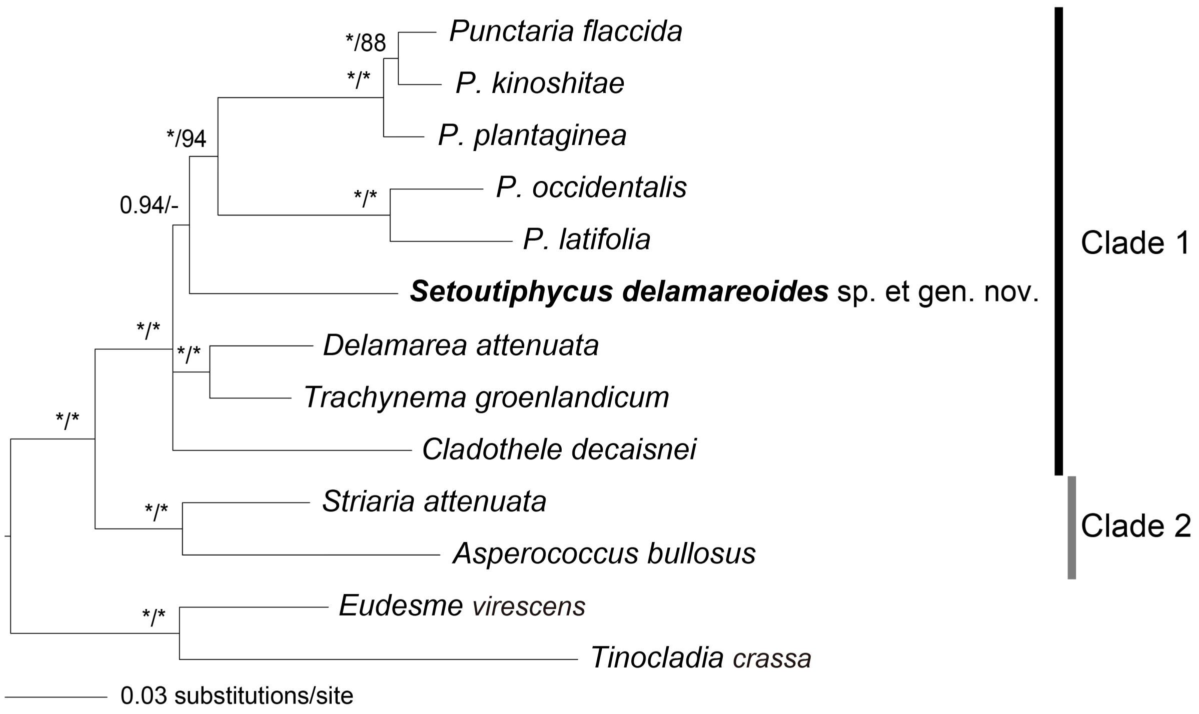

Bayesian consensus tree based on the concatenated DNA sequences of mitochondrial *cox*1, *cox*3, *nad*2, *nad*5 and *nad*6 genes, and chloroplast *atp*B, *psa*A, *psb*A and *rbc*L genes (total 10,975 bp). Numbers on branches indicate posterior probabilities from Bayesian analysis (left) and bootstrap values (%) from ML analysis (right). Asterisk (*) indicates 1.00 posterior probability (Bayesian) and 100% bootstrap (ML) values. Only posterior probabilities ≥ 0.90 and bootstrap values ≥ 70% are shown.

Supplementary Information 6

p-distance among genera within clade 1 in Figure 3 based on *rbc*L (upper diagonal) and *cox*3 (lower diagonal) sequences.

	Setoutiphycus	*Asperococcus*	*Cladothele*	*Delamarea*	*Punctaria*	*Striaria*	*Trachynema*
Setoutiphycus	-	0.027	0.029	0.014	0.018	0.022	0.017
Asperococcus	0.104	-	0.038	0.023	0.031	0.014	0.025
Cladothele	0.098	0.110	-	0.023	0.032	0.030	0.028
Delamarea	-	-	-	-	0.019	0.016	0.010
Punctaria	0.097	0.104	0.101	-	-	0.026	0.018
Striaria	0.079	0.064	0.100	-	0.094	-	0.019
Trachynema	0.074	0.088	0.084	-	0.092	0.074	-

**Delamarea* was not included in the analysis of *cox*3 sequences, because the available *cox*3 sequence was partial (157 bp).

Supplementary Information 7

Genetic distances (p-distance) of mitochondrial *cox*3 gene between selected ectocarpalean and laminarialean genera.

Compared genera	p-distance	Reference
**Ectocarpales**		
Setoutiphycus vs. Asperococcus, Cladothele, Delamarea, Punctaria, Striaria or Trachynema	0.074–0.104	This study
Cladosiphon vs. *Mesogloia*	0.091–0.144	Kawai *et al.* (2019a)
Dictyosiphon vs. *Asperococcus* or *Punctaria*	0.103–0.131	Kawai *et al.* (under revision)
Eudesme vs. *Tinocladia*	0.0960.117	Hanyuda *et al.* (2019), Kawai *et al.* (2019b)
Saundersella vs. *Acrothrix* or *Cladosiphon*	0.116-0.128	Kawai et al. (2021)
**Laminariales**		
Agarum vs. *Costaria*, *Dictyoneurum*, or *Neoagarum*	0.042–0.064	Kawai *et al.* (2017)
Ecklonia vs. *Eisenia*	0.024-0.043	Kawai *et al.* (2020)
Saccharina vs. *Nereocystis*	0.094–0.106	Augyte *et al.* (2018), Yotsukura *et al.* (2010), Zheng *et al.* (2013, 2019)

References of Supplementary Information 7.

Augyte, S., Lewis, L., Lin, S., Neefus, C. D. & Yarish, C. Speciation in the exposed intertidal zone: the case of *Saccharina angustissima comb. nov. & stat. nov.* (Laminariales, Phaeophyceae). *Phycologia* **57**, 100–112 (2018).

Hanyuda, T., Takeuchi, T. & Kawai, H. *Tinocladia sanrikuensis* sp. nov. (Ectocarpales *s.l.*, Phaeophyceae) from Japan. *Phycol. Res.* **67**, 221–227. (2019).

Kawai, H., Hanyuda, T., Gao, X., Terauchi, M., Miyata, M., Lindstrom, S. C., Klochkova, N. G. & Miller, K. A. Taxonomic revision of the Agaraceae with a description of *Neoagarum gen. nov.* and reinstatement of *Thalassiophyllum. J. Phycol.* **53**, 261–270 (2017).

Kawai, H., Hanyuda, T., Shibata, K., Kamiya, M. & Peters, A. F. Proposal of a new brown algal species *Mesogloia japonica* sp. nov. (Chordariaceae, Phaeophyceae) and transfer of *Sauvageaugloia ikomae* to *Mesogloia. Phycologia* **58**, 63–69 (2019a).

Kawai, H., Hanyuda, T., Sun, Z. M, Bárbara, I. & Peters, A.F. Taxonomic revision of *Eudesme* (Ectocarpales *s.l.*, Phaeophyceae) proposing a new species *E. borealis* sp. nov. *Phycologia* **58**, 351–358 (2019b).

Kawai, H., Akita, S., Hashimoto, K. & Hanyuda, T. A multigene molecular phylogeny of *Eisenia* reveals evidence for a new species, *Eisenia nipponica* (Laminariales), from Japan. *Europ. J. Phycol.* **55**, 234–241 (2020).

Kawai, H., Watanabe, Y., Kawai, H. Taxonomic revision of *Saundersella* (Ectocarpales *s.l.,* Phaeophyceae) with description of *Saundersella crassa sp. nov*. and transfer of *Heterosaundersella hattoriana* to *Saundersella. Phycologia* published online. (2021). 10.1080/00318884.2021.1916859

Yotsukura, N., Shimizu, T., Katayama, T. & Druehl, L. D. Mitochondrial DNA sequence variation of four Saccharina species (Laminariales, Phaeophyceae) growing in Japan. *J Appl. Phycol.* **22**, 243–251 (2010).

Zheng, J., Wang, X., Liu, C., Jin, Y. & Liu, T. The complete mitochondrial genomes of two brown algae (Laminariales, Phaeophyceae) and phylogenetic analysis within Laminaria. *J Appl. Phycol.* **25**, 1247–1253 (2013).

Zheng, Z., Chen, H., Wang, H., Jiang, W., Cao, Q. & Du, N. Characterization of the complete mitochondrial genome of bull kelp, *Nereocystis luetkeana*. Mitochondrial DNA B Part B **4**, 630–631 (2019).

Supplementary Information 8

Sequence divergence (p-distance) of chloroplast *rbc*L genes among representative genera within selected brown algal orders.

Genera compared within each order / number of genera in each order	p-distance	Species and DNA database codes
**Dictyotales** (*Dictyota, Exallosorus, Padina* and *Stypopodium*) / 20	–0.127	*Dictyota crenulata* (GU290253)<br>*Exallosorus olsenii* (DQ866923)<br>*Padina japonica* (AB358910)<br>*Stypopodium schimperi* (DQ866926)
**Ectocarpales *s.l.*** (*Acinetospora*, *Adenocystis*, Ectocarpus, *Petrospongium, Scytosiphon, Setoutiphycus* and *Stictyosiphon*) / 139	–0.092	*Acinetospora asiatica* (LC060517)<br>*Adenocystis utricularis* (AJ295823)<br>*Ectocarpus* sp. (FP102296)<br>*Petrospongium rugosum* (AY996361)<br>*Scytosiphon lomentaria* (AB022238)<br>*Setoutiphycus delamareoides* (LC603808)<br>*Stictyosiphon soriferus* (AB775343)
**Fucales** (*Fucus*, *Hormosira, Phyllospora* and *Sargassum*) /50	–0.101	*Fucus vesiculosus* (FM957154)<br>*Hormosira banksii* (AB776774)<br>*Phyllospora comosa* (EF990249)<br>*Sargassum muticum* (AB776776)
**Ishigeales** (*Diplura*, *Ishige* and *Petroderma*) / 3	–0.163	*Diplura simplex* (AB250084)<br>*Ishige okamurae* (AY372974)<br>*Petroderma maculiforme* (EU579934)
**Chordales** (*Akkesiphycus, Chorda* and *Pseudochorda*) / 3	–0.080	*Akkesiphycus lubricum* (AB036038)<br>*Chorda asiatica* (AB775335)<br>*Pseudochorda nagaii* (AB775342)
**Laminariales** (*Aureophycus*, *Macrocystis*, *Neoagarum* and *Undaria*) / 33	–0.082	*Aureophycus aleuticus* (AB355025)<br>*Macrocystis pyrifera* (AB775339)<br>*Neoagarum oharaense* (LC148138)<br>*Undaria pinnatifida* (AB775334)
**Sphacelariales** (*Bodanella*, Halopteris, *Phaeostrophion* and *Sphacelaria*) / 10	–0.123	*Bodanella lauterborni* (FM956110)<br>*Halopteris gracilescens* (AB899291)<br>*Phaeostrophion irregulare* (AB899292)<br>*Sphacelaria radicans* (FM956112)
**Sporochnales** (*Bellotia*, *Carpomitra* and *Sporochnus*) / 11	–0.057	*Bellotia eriophorum* (AB899295)<br>*Carpomitra costata* (AB776780)<br>*Sporochnus scoparius* (AB776781)
**Stschapoviales** (*Halosiphon, Platysiphon* and *Stschapovia*) / 3	–0.057	*Halosiphon tomentosus* (AB545977)<br>*Platysiphon glacialis* (AB776772)<br>*Stschapovia flagellaris* (AB117920)
**Tilopteridales** (*Cutleria, Saccorhiza* and *Tilopteris*) / 7	–0.077	*Cutleria adspersa* (AB545967)<br>*Saccorhiza polyschides* (AB545978)<br>*Tilopteris mertensii* (AB776784)
